# Study on the disinfection effect of chlorine dioxide disinfectant (ClO_2_) on dental unit waterlines and its in vitro safety evaluation

**DOI:** 10.1186/s12903-024-04391-7

**Published:** 2024-06-01

**Authors:** Cao Yue, Hu Yuya, Liu Zhihuan, Wang Zimo, Feng Jianying

**Affiliations:** School of Stomatology, Zhejiang Chinese Medical University, Hangzhou, Zhejiang, 310053 China

**Keywords:** Chlorine dioxide, Disinfectant, Dental equipment waterlines, Antimicrobial, Metal corrosion, Cytotoxicity

## Abstract

**Background:**

Ensuring the safety of dental unit waterlines (DUWLs) has become a pivotal issue in dental care practices, focusing on the health implications for both patients and healthcare providers. The inherent structure and usage conditions of DUWLs contribute to the risk of biofilm formation and bacterial growth, highlighting the need for effective disinfection solutions.The quest for a disinfection method that is both safe for clinical use and effective against pathogens such as *Staphylococcus aureus* and *Escherichia coli* in DUWLs underscores the urgency of this research.

**Materials:**

Chlorine dioxide disinfectants at concentrations of 5, 20, and 80 mg/L were used to treat biofilms of *S. aureus* and *E. coli* cultured in DUWLs. The disinfection effectiveness was assessed through bacterial counts and culturing. Simultaneously, human skin fibroblast cells were treated with the disinfectant to observe changes in cell morphology and cytotoxicity. Additionally, the study included corrosion tests on various metals (carbon steel, brass, stainless steel, aluminum, etc.).

**Results:**

Experimental results showed that chlorine dioxide disinfectants at concentrations of 20 mg/L and 80 mg/L significantly reduced the bacterial count of *S. aureus* and E. coli, indicating effective disinfection. In terms of cytotoxicity, higher concentrations were more harmful to cellular safety, but even at 80 mg/L, the cytotoxicity of chlorine dioxide remained within controllable limits. Corrosion tests revealed that chlorine dioxide disinfectants had a certain corrosive effect on carbon steel and brass, and the degree of corrosion increased with the concentration of the disinfectant.

**Conclusion:**

After thorough research, we recommend using chlorine dioxide disinfectant at a concentration of 20 mg/L for significantly reducing bacterial biofilms in dental unit waterlines (DUWLs). This concentration also ensures satisfactory cell safety and metal corrosion resistance.

## Introduction

Microbial contamination of DUWLs has been a significant concern in the field of dental treatment [1]. Due to their unique design and usage patterns, DUWLs are prone to the formation of biofilms within their internal pipelines [2–4]. These biofilms serve as breeding grounds for various microorganisms, including potentially harmful bacteria such as *Staphylococcus aureus* and *Escherichia coli*, which may pose threats to human health [5]. The presence of these microorganisms not only has the potential to impact the quality and safety of dental treatments but also poses health risks to both patients and healthcare personnel through cross-contamination [6–8]. Several methods and disinfectants have been proposed and employed for the disinfection of DUWLs [9–11], ranging from traditional chemical agents to advanced techniques like plasma sterilization and nanometer silver (NMS) [12, 13].

Chlorine dioxide [14], known for its proven high germicidal capabilities, has been widely utilized in various industries. Its effectiveness in disinfecting a wide range of microorganisms, including bacteria, viruses, spores, and fungi, has been demonstrated in numerous studies. Nevertheless, the existing research data on the application of chlorine dioxide in the dental field, particularly in DUWLs disinfection, is relatively limited, and its safety concerning human cells and its corrosiveness to DUWLs materials require further investigation.The primary aim of this research is to thoroughly assess the efficacy of chlorine dioxide disinfectants in DUWLs, focusing on their ability to eliminate key pathogens, such as *Staphylococcus aureus* and *Escherichia coli*, and their impact on both human cell viability and equipment integrity. Emphasizing a balanced approach, this study investigates the potential of chlorine dioxide not only as a potent antimicrobial agent but also in terms of its compatibility with dental practice requirements, ensuring patient and equipment safety. This holistic evaluation seeks to establish a clear, scientific basis for the optimal use of chlorine dioxide in dental settings, addressing concerns over microbial resistance and environmental hazards while promoting a safer healthcare environment.

## Materials and methods

### Preparation of disinfectant solutions for experimental use

To prepare the disinfectant stock solutions, we dissolved chlorine dioxide effervescent tablet (Basteur, China) in sterile water to create a 100 mg/L solution, and similarly, we dissolved chlorine-containing effervescent tablet (Health Essence, China) in sterile water to achieve a 250 mg/L solution. We then diluted these stock solutions to obtain various concentrations of chlorine dioxide solutions (5, 20, 80 mg/L) and a 20 mg/L chlorine-containing disinfectant solution for experimental use.

### In vitro antimicrobial performance evaluation

At the onset of the experiment, single colonies were inoculated into 20 mL of nutrient broth and incubated on a shaker at 220 rpm and 37 °C for 24 h. Afterwards, a sample of the culture was mixed with an equal volume of sterile water, and its OD405 value was measured using a spectrophotometer. Concurrently, DUWLs tubes (Sinol，China) were segmented into 100 test tubes, each with an inner diameter of 2 mm and a length of 21 mm, for cultivating biofilms of *Staphylococcus aureus* (S. aureus, ATCC 25,923) and *Escherichia coli* (E. coli, ATCC 25,922). These tubes were placed in centrifuge tubes containing 1 mL of nutrient agar and 100 µL of bacterial solution, and incubated at 37 °C for 48 h.

Subsequently, chlorine dioxide solutions of varying concentrations (5, 20, 80 mg/L) and a 20 mg/L chlorine-containing disinfectant were prepared. Biofilm samples of the two bacterial strains were divided into five groups (nine samples per group), with experimental groups treated with different concentrations of chlorine dioxide solution for 15 min and control groups treated with chlorine-containing disinfectant and physiological saline in the same manner. The treated water pipe samples were rinsed with 2 ml of saline; the outer surface of the pipe wall was wiped with alcohol wipes to remove microorganisms from the outer wall of the pipeline; the water pipe samples were cut horizontally into four parts, placed in test tubes, and 10 ml of saline was added. The tubes were sealed and shaken in an ultrasonic shaker for 15 min. Using aseptic techniques, 100 µL of the sample was inoculated onto solidified nutrient agar medium and evenly spread. The Petri dishes were then incubated in a constant temperature incubator at 36 °C ± 1 °C for 48 h. Finally, bacterial counts were calculated and reported according to the GB/T 5750.12.2006 standard, with units in CFU/mL.

### Cell safety assessment

#### Cell morphology observation

Ninety-six-well plates were used for cell morphology observation. Human dermal fibroblast cells(HDF)were purchased from Shanghai Xinyu Biotechnology Co., Ltd., and cells from the 3rd to 7th passage were selected for experiments. Four experimental groups were designated according to the concentrations of the disinfectants used (chlorine dioxide at 5, 20, and 80 mg/L, and a 20 mg/L chlorine-based disinfectant), accompanied by a blank control group. Cells were plated at a density of 4 × 10^4 cells/mL and cultured under conditions of 37℃ and 5% CO_2_ for 24 h. Cells were cultured for 2 h and 24 h under the same conditions, and cell morphology was observed using an inverted microscope.

#### CCK-8 assay

Twenty-four hours after adding the various concentrations of disinfectants, the liquid from the wells of both the experimental and control groups was removed, and the wells were washed twice with sterile PBS. A CCK-8 working solution was prepared at a volume ratio of 1:10. Each well of the blank, control, and experimental groups was then filled with 110uL of the CCK-8 working solution. The 96-well plate was placed in a cell culture incubator, protected from light, and incubated for 3 h. The absorbance (OD value) was measured at a wavelength of 450 nm using a microplate spectrophotometer, ensuring that there were no bubbles in each well before measurement.

### Metal corrosion testing

Prior to the experiment, four experimental groups were established based on disinfectant concentrations (5, 20, 80 mg/L chlorine dioxide and 20 mg/L chlorine-containing disinfectant), along with one blank control group. Circular discs were prepared from four types of metals (carbon steel, aluminum, copper, stainless steel) in accordance with the specifications set forth in the 2002 Disinfection Technology Standards. Each disc featured a thickness of 1.0 mm, a diameter ranging from 23.9 mm to 24.1 mm, and a central hole with a diameter of 2.0 mm. Initial preparation involved removing the oxide layer, degreasing, drying, and weighing the discs. Subsequently, the discs were immersed in varying concentrations of disinfectants, with three discs per metal type, each in a separate container. The disinfectant solution was replaced daily. For the control group, discs were immersed in sterile water. After 72 h of immersion, the metal discs were removed, cleaned of corrosion products, dried, and reweighed. The corrosion rate (R) of each disc was then calculated. Metals with significant corrosion were selected for surface analysis via laser confocal scanning to examine the extent of corrosion. The corrosion rate of the metal can be calculated using the following formula:

$$R=\frac{{8.76\times 10}^{7}\times \left(m-mt-mk\right)}{S\times t\times d}$$

R is the corrosion rate of the metal, measured in millimeters per year (mm/a).

m is the initial weight before the test, measured in grams (g). mt is the weight after the test, measured in grams (g). mk is the weight loss after chemical cleaning, measured in grams (g). S is the surface area of the metal, measured in square centimeters (cm²).t is the treatment time, measured in hours (h). d is the density of the sample, measured in kilograms per cubic meter (kg/m³).

### Statistical analysis

Statistical analysis was carried out using SPSS 25.0.A one-way analysis of variance (ANOVA) was conducted followed by using Tamhane’s post hoc test to assess the disinfection effects in different groups. A p-value less than 0.05 was considered statistically significant. Additionally, dimensional plots and the Shapiro-Wilk test were used to evaluate normality using GraphPad Prism. One-way analysis of variance (ANOVA) was conducted to assess the influence of various disinfectants on cell viability, with α set at 0.05 for all tests.

## Results

### In vitro antimicrobial activity assessment

**Fig. 1:**
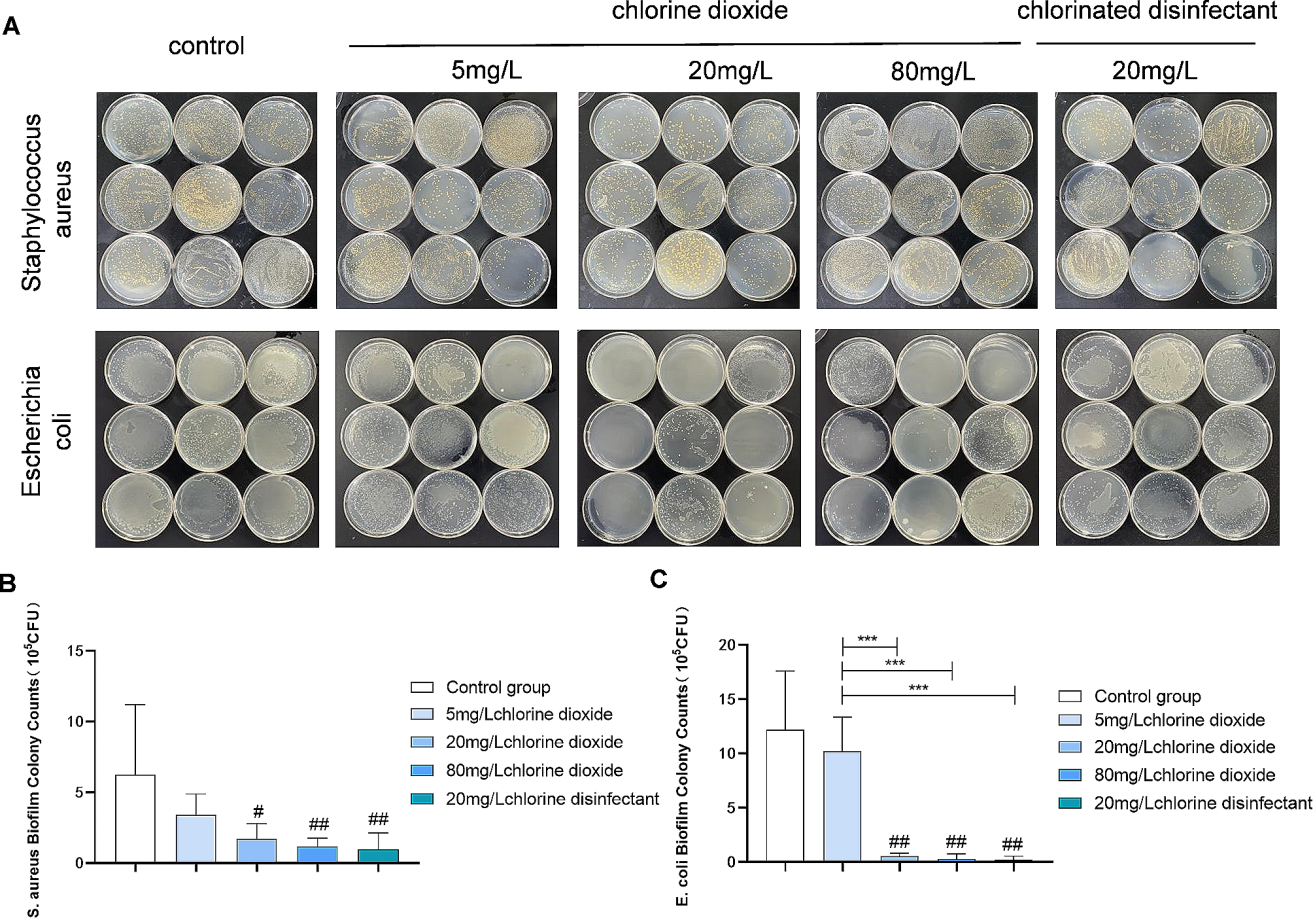
Evaluation of antibacterial effect of different disinfectants. Staphylococcus aureus and Escherichia coli cultures on agar plates (**A**) and colony analysis (**B**) (**C**) show the effective antibacterial property in the group of 20/80 mg/L chlorine dioxide and 20 mg/L chlorine-containing disinfection

This study examines the effects of different disinfectant concentrations on the clearance of *Staphylococcus aureus* and *Escherichia coli* biofilms. Significant reductions in bacterial counts for *Staphylococcus aureus* were observed with 20 mg/L and 80 mg/L chlorine dioxide, as well as with a 20 mg/L chlorine-containing disinfectant, highlighting their effectiveness in biofilm clearance(Fig. 1A). However, comparisons between the experimental groups themselves did not show statistical significance(Fig. 1B)., indicating that while all treatments were effective compared to the control, they were similarly effective when compared to each other. Similarly, all concentrations tested, except for 5 mg/L chlorine dioxide, significantly reduced *Escherichia coli* biofilm bacterial counts, with no significant difference among the higher concentrations, indicating a plateau in efficacy beyond 20 mg/L. Statistical analyses confirm these outcomes as significant (*p* < 0.05).

The asterisks denote the level of statistical significance, i.e., comparison with control group, ^#^: *P* < 0.05, ^##^:*P* < 0.01; comparison among groups *: *P* < 0.05, **: *P* < 0.01, *** :*P* < 0.001.

### Cell morphology and viability analysis

**Fig. 2:**
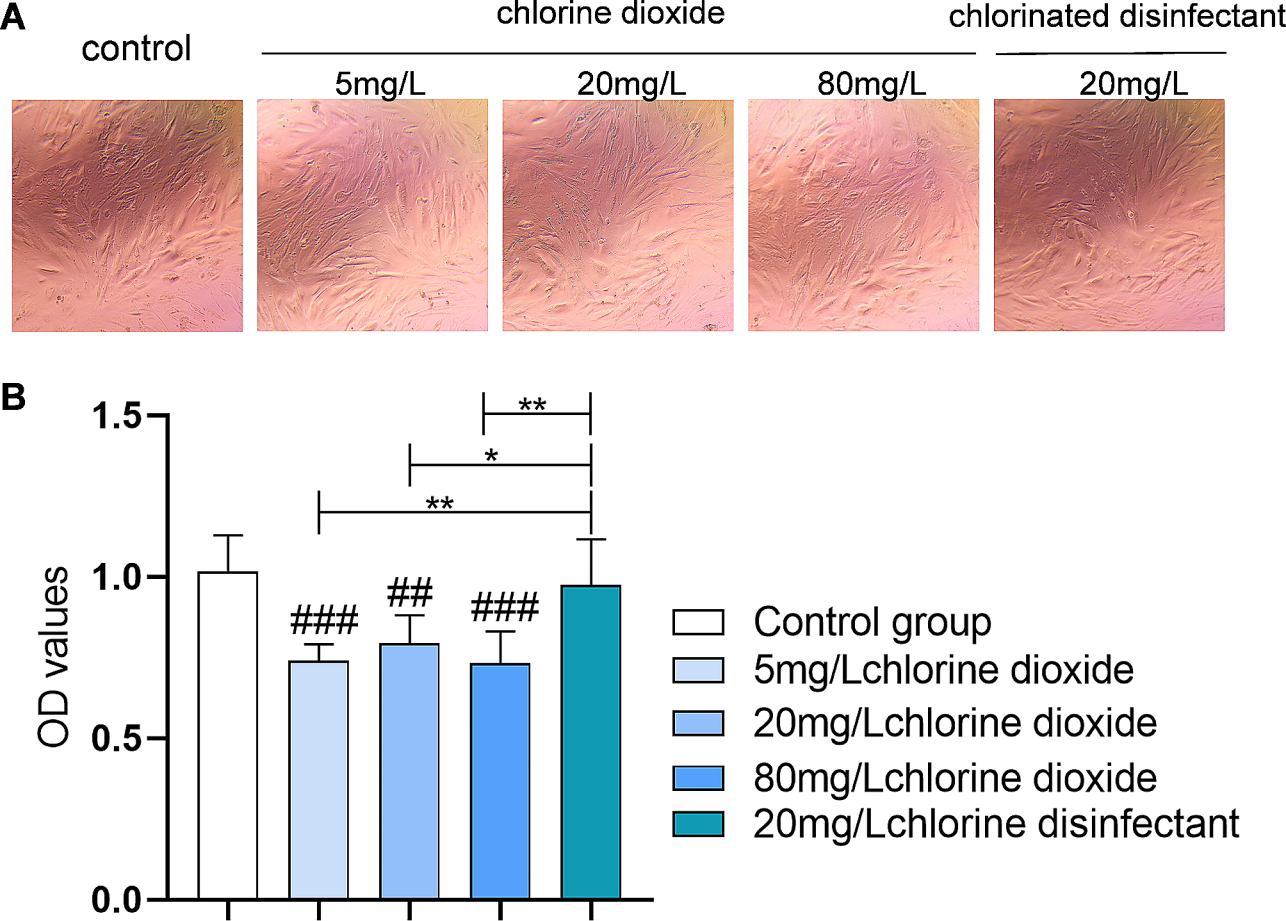
The cytotoxicity impact of chlorine dioxide (5, 20, 80 mg/L) and chlorinated disinfectants (20 mg/L) on human skin fibroblast cells. (**A**) Cell Morphology and (**B**) viability analysis after 24 h of exposure to different disinfectants shows with the increasing concentrations of chlorine dioxide disinfectant, the cytotoxicity also increases

The impact of chlorine dioxide and chlorinated disinfectants on human skin fibroblast cells was assessed through morphological observations under an inverted microscope and cytotoxicity evaluation using a CCK-8 assay. After 24-hour exposure to 5, 20, 80 mg/L chlorine dioxide and 20 mg/L chlorinated disinfectant, cell morphology changes were notable (Fig. 2A). At 5 mg/L chlorine dioxide, cells retained their normal morphology, indicating minimal toxicity. A moderate concentration of 20 mg/L began to induce slight morphological alterations and mild toxicity, while at 80 mg/L, significant changes in cell shape and reduced adherence were observed, suggesting increased cytotoxic effects. Conversely, the 20 mg/L chlorinated disinfectant exhibited milder impacts on cell morphology, suggesting lower or comparable toxicity to the medium concentration of chlorine dioxide. This suggests that with increasing concentrations of chlorine dioxide disinfectant, its cytotoxicity also increases. This gradation in cytotoxicity with increasing chlorine dioxide concentration was quantitatively supported by the CCK-8 assay, where the absorbance values indicated the relative cell viability and disinfectant toxicity. Additionally, the chlorinated disinfectant appeared to have better cell compatibility, as supported by the statistical analysis presented in Fig. 2 (B). However, the overall higher absorbance values suggest that the disinfectants’ usage concentrations might be within safe limits for the cells, as further supported by statistical analysis in Fig. 2.

The asterisks denote the level of statistical significance, i.e., comparison with control group, ^##^:*P* < 0.01, ^###^ :*P* < 0.001; comparison among groups *: *P* < 0.05, **: *P* < 0.01.

### Metal corrosion resistance test

Prior to the main study, our team conducted preliminary experiments, revealing that stainless steel and aluminum exhibited lower corrosion resistance. Consequently, we directed our research towards assessing the corrosive impact of disinfectants on carbon steel and brass. The initial tests indicated that corrosion severity increased with higher disinfectant concentrations. For enhanced clarity under the electron microscope, we excluded the less corrosive control group and the 5 mg/L chlorine dioxide condition, focusing instead on 20 mg/L and 80 mg/L concentrations to explore the correlation between disinfectant strength and corrosion intensity.

Our results demonstrate that the corrosion rate escalates with the concentration of chlorine dioxide. Carbon steel showed a range of corrosion from mild to moderate across different concentrations, whereas brass exhibited relatively less corrosion. These outcomes, quantified and tabulated (Table 1), facilitate a direct comparison of the susceptibility of the two metals to chlorine dioxide. Additionally, it is crucial to compare these results with those involving chlorinated disinfectants. Our findings suggest that chlorinated disinfectants induce a more pronounced corrosive effect on both metals than chlorine dioxide, highlighting its superior resistance to metal corrosion. This distinction emphasizes the advantages of chlorine dioxide over traditional chlorinated disinfectants, particularly in applications where reducing metal corrosion is essential.

**Table 1:** Corrosion rates of carbon steel and brass in various disinfectants

Disinfectant	Carbon steel	Brass
	Corrosion rate (*R*) [mm/year]	Corrosion rate (*R*) [mm/year]
Control Group	0.05278 (R2)	0.00243 (R1)
5 mg/L Chlorine Dioxide	0.09659 (R2)	0.00973 (R1)
20 mg/L Chlorine Dioxide	0.10556 (R2)	0.02336 (R2)
80 mg/L Chlorine Dioxide	0.28503 (R3)	0.03602 (R2)
20 mg/L Chlorinated Disinfectant	0.32462 (R3)	0.01509 (R2)

The corrosion rates are classified as follows: Rates below 0.05 mm/year indicate no corrosion (R0); rates between 0.005 and 0.01 mm/year suggest negligible corrosion (R1); rates greater than 0.01 but up to 0.1 mm/year are indicative of mild corrosion (R2); rates over 0.1 up to 1.0 mm/year represent moderate corrosion (R3); and rates exceeding 1.0 mm/year denote severe corrosion (R4). The values in parentheses (R1, R2, R3) beside corrosion rates denote the replicates for each measurement.

**Fig. 3:**
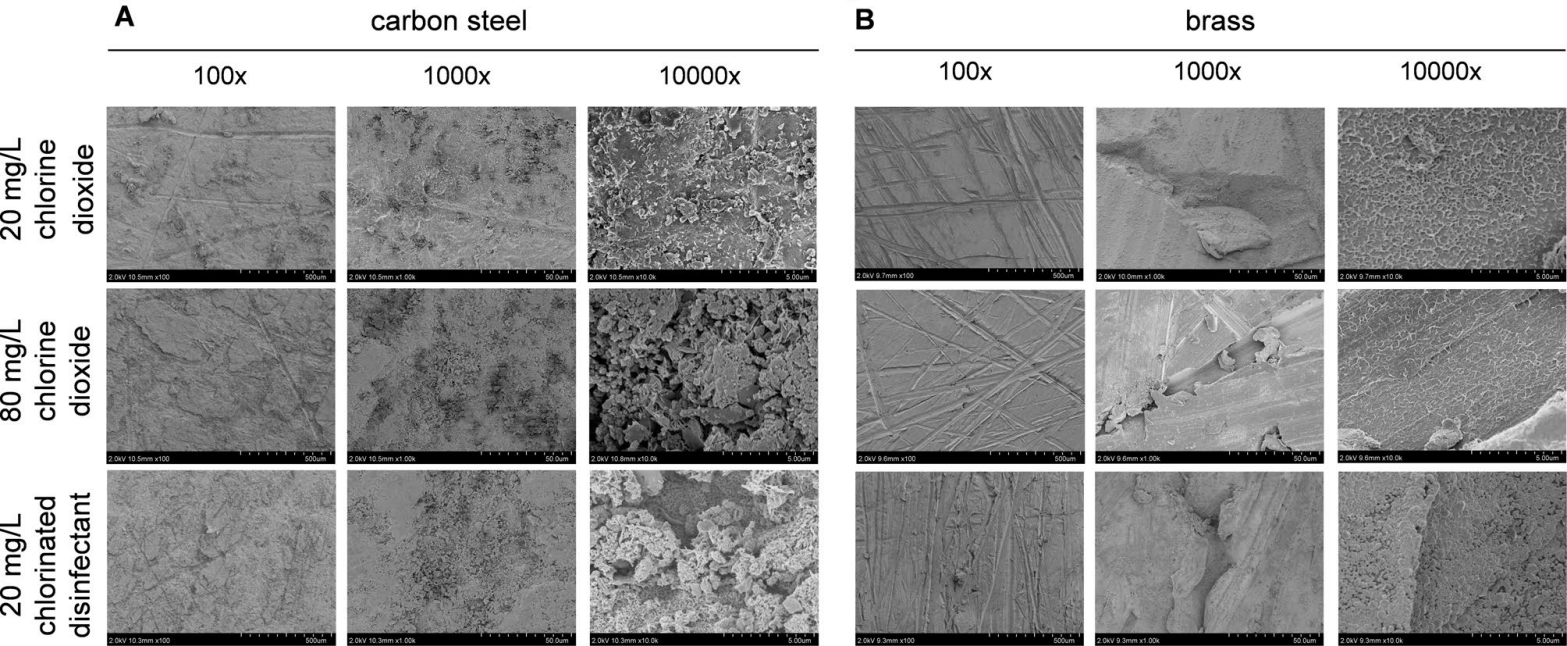
Scanning Electron Microscopy (SEM) observation of carbon steel (**A**) and brass (**B**) after 72 h of immersion in the different disinfectants. Chlorinated disinfectants(20 mg/L) induce a more pronounced corrosive effect on both metals than chlorine dioxide (20/80 mg/L)

The images display scattered black dot structures on samples treated with 20 mg/L chlorine dioxide disinfectant. Increasing the concentration to 80 mg/L results in a rough, corrosive morphology marked by numerous punctate cracks and corrosion pits. Conversely, samples with 20 mg/L chlorinated disinfectant show small pit-like corrosions but no fractures. SEM analysis reveals that corrosion is more severe in carbon steel compared to brass. Specifically, SEM images of carbon steel (Fig. 3A) illustrate extensive damage with detailed views of rough surfaces, cracks, and pits. In contrast, SEM images of brass (Fig. 3B) demonstrate minimal pit-like corrosions, providing a comparative perspective on the materials’ resilience to disinfectant exposure. The analysis indicates that while both disinfectants at 20 mg/L induce corrosion, the chlorinated disinfectant has a stronger corrosive effect. This comparison highlights the superior corrosion resistance of chlorine dioxide, particularly in maintaining the long-term integrity of metal surfaces.

## Discussion

The daily disinfection of DUWLs has been paid more and more attention in dental clinic, especially after the pandemic [15–17]. At present, the most commonly used disinfectant is 20 mg/L chlorine-containing disinfectant, but there are hidden problems such as incomplete disinfection, metal corrosion and bio-safety for long-term use [17, 18]. Chlorine dioxide can be used effectively for broad spectrum sterilization, including bacteria, viruses, spores, and fungi. In this study, we investigated the properties of chlorine dioxide from the aspects of antibacterial, cytotoxicity [19] and metal corrosion [20]. The experimental results show that chlorine dioxide solution with appropriate concentration can be used as an effective substitute for disinfectant in DUWLs.

The optimal concentration of 20 mg/L chlorine dioxide was found to be the most effective across multiple evaluation criteria, including bacterial disinfection, cytotoxicity to human cells, and corrosion resistance to metals [16, 21–25]. Moreover, comparative analysis highlighted ClO_2_’s safer profile at regulated concentrations compared to 20 mg/L chlorinated disinfectant, with lower inhibitory effects on cell viability. This aligns with broader research advocating for ClO_2_’s role in minimizing the formation of harmful disinfection by-products (DBPs) such as chlorite and chlorate, which are linked to potential health risks, while effectively controlling waterborne pathogens [26]. This integrated approach underscores the need for a balanced disinfection strategy DUWLs, optimizing microbial kill rates while reducing cytotoxicity and corrosion resistance.

Although it performs similarly to other chlorine-containing disinfectants in some respects, 20 mg/L chlorine dioxide is distinguished by its overall utility, making it a preferred choice for disinfecting dental chair waterways [27–29]. Indeed, we have initiated preliminary clinical trials to evaluate the effectiveness of 20 mg/L chlorine dioxide as a disinfectant within clinical settings. The early outcomes are promising, showing a positive impact on safety and infection control. Moving forward, we will continue our research to enhance safety and reduce infection risks in dental care environments. Additional studies are essential to determine the practicality of this concentration and refine its application for broader integration into dental health practices. Furthermore, exploring the unique properties of 20 mg/L chlorine dioxide that confer these advantages remains a critical area of our ongoing research, aiming to fully understand its efficacy and mechanism in various dental care scenarios.

## Conclusion

Chlorine dioxide disinfectant optimally balances efficacy, safety, and cost in disinfecting DUWLs. At a concentration of 20 mg/L, it effectively reduces microbial contamination with minimal cytotoxicity and equipment corrosion, presenting a superior alternative for dental water systems. This concentration demonstrates enhanced safety and economic benefits over traditional methods, advocating for its broader use in dental practices.

Future research should further explore optimal application conditions for 20 mg/L chlorine dioxide in dentistry. With its low toxicity and mild corrosiveness, it holds potential for enhancing safety and reducing infection risks. Our study strengthens the case for chlorine dioxide’s use in dentistry, paving the way for future research to refine disinfection protocols and prolong dental equipment lifespan.

## Data Availability

All data generated or analysed during this study are included in this published article.
